# *Lactobacillus rhamnosus* Accelerates Zebrafish Backbone Calcification and Gonadal Differentiation through Effects on the GnRH and IGF Systems

**DOI:** 10.1371/journal.pone.0045572

**Published:** 2012-09-20

**Authors:** Matteo A. Avella, Allen Place, Shao-Jun Du, Ernest Williams, Stefania Silvi, Yonathan Zohar, Oliana Carnevali

**Affiliations:** 1 Department of Life and Environmental Sciences, Polytechnic University of Marche, Ancona, Italy; 2 Institute of Marine and Environmental Technology, University of Maryland, Center of Environmental Sciences, Baltimore, Maryland, United States of America; 3 Department of Biochemistry & Molecular Biology, University of Maryland School of Medicine, Baltimore, Maryland, United States of America; 4 School of Bioscience and Biotechnology, University of Camerino, Camerino, Italy; 5 Institute of Marine and Environmental Technology & Department of Marine Biotechnology, University of Maryland Baltimore County, Baltimore, Maryland, United States of America; Universitat Pompeu Fabra, Spain

## Abstract

Endogenous microbiota play essential roles in the host’s immune system, physiology, reproduction and nutrient metabolism. We hypothesized that a continuous administration of an exogenous probiotic might also influence the host’s development. Thus, we treated zebrafish from birth to sexual maturation (2-months treatment) with Lactobacillus rhamnosus, a probiotic species intended for human use. We monitored for the presence of L. rhamnosus during the entire treatment. Zebrafish at 6 days post fertilization (dpf) exhibited elevated gene expression levels for Insulin-like growth factors -I and -II, Peroxisome proliferator activated receptors -α and -β, VDR-α and RAR-γ when compared to untreated-10 days old zebrafish. Using a gonadotropin-releasing hormone 3 GFP transgenic zebrafish (GnRH3-GFP), higher GnRH3 expression was found at 6, 8 and 10 dpf upon L. rhamnosus treatment. The same larvae exhibited earlier backbone calcification and gonad maturation. Noteworthy in the gonad development was the presence of first testes differentiation at 3 weeks post fertilization in the treated zebrafish population -which normally occurs at 8 weeks- and a dramatic sex ratio modulation (93% females, 7% males in control vs. 55% females, 45% males in the treated group). We infer that administration of L. rhamnosus stimulated the IGF system, leading to a faster backbone calcification. Moreover we hypothesize a role for administration of L. rhamnosus on GnRH3 modulation during early larval development, which in turn affects gonadal development and sex differentiation. These findings suggest a significant role of the microbiota composition on the host organism development profile and open new perspectives in the study of probiotics usage and application.

## Introduction

The group of Lactic acid bacteria (LAB) represents a large part of the microbiota of vertebrates [1] and their beneficial effects on the immune system [2]–[4], gastrointestinal tract [5], and reproduction [6], [7] have been widely reported. Moreover, studies with germ free mice [8] and zebrafish [9] have shown that host microbiota can modify host nutrient metabolism and energy balance [10]. Due to a distinct selective pressure imposed within the host’s gut habitat, the microbiota community structure is different between zebrafish and mice [11]. We speculated that different microbiota compositions might correspond to different physiological and developmental performances; thus, in the current study we wondered what effects we could induce in the developmental profile of the host organism if we influenced the gut microbiota from birth to puberty by providing a selected LAB strain. We chose zebrafish as our model because of its short life cycle, well documented gonad developmental profile and the fact that the calcification process of the skeleton has been previously described [12]. The LAB strain we selected was Lactobacillus rhamnosus, which is one of the main components of the commensal microflora of human intestinal tract of and is widely used as a probiotic in mammals [13]–[15].

Upon L. rhamnosus treatment, quantitative gene expression of a number of markers involved in stress response usually induced by captive conditions, muscle growth and development were monitored, together with growth rate of the larvae. Stress is one of the main causes affecting growth with cortisol being the primary mediator for stress response in fish; cortisol effects are mediated by the intracellular glucocorticoid receptor (GR) and GR mRNA abundance is correlated with circulating cortisol levels [16]. Together with GR, 70-kDa heat shock protein (HSP70) has been widely used as a biomarker for stress response to captive conditions [17]–[19]. Therefore, we used GR and HSP70 gene expression to evaluate stress status upon L. rhamnosus treatment. Eventually stress can impact the whole animal’s performance, in terms of growth and metabolism [20]. Fish growth is positively correlated to muscle growth and is controlled by extrinsic regulators such as insulin-like growth factor-1 (IGF-1), insulin-like growth factor-2 (IGF-2) and myostatin (MSTN), which are involved in fish myogenesis [21]–[23] and have a role in muscle growth [24]. Moreover, gene expression of IGF-1 and IGF-2 are dependent on feeding regime [25]. MSTN, a member of the transforming growth factor-β (TGF- β) superfamily, acts in an opposite way by inhibiting muscle cell proliferation [26], [27]. We tracked IGFs and MSTN gene expression to see whether L. rhamnosus administration could affect growth by positively acting on the IGF system. Indeed, growth metabolism is correlated to several other factors, some of which belong to the nuclear receptor superfamily. Peroxisome proliferator activated receptors (PPARs), retinoic acid receptors (RARs) and vitamin D receptors (VDRs), are natural receptors for many organic compounds delivered by LAB activity and modulate optimal growth and correct development [28]. These factors affect and regulate growth and organism remodelling through processes such as lipid metabolism [29], energy administration and epithelial cell growth and differentiation [30], calcium homeostasis, growth and bone formation [31], morphogenesis and chondrogenesis [32]. PPAR-α and -β are known to be involved in lipid metabolism and cell growth [33], [34] and to be activated by fatty acids [35], [36]. For example, LAB fermentation products, including short-chain fatty acids and immunoregulatory molecules as eicosanoids, are known to activate PPARs [29] and for these reasons we used PPAR-α and -β gene expression as biomarkers to evaluate the effects of L. rhamnosus on lipid production.

VDRs mediate the effects of the most bioactive derivative of vitamin D, 1,25-dihydroxyvitamin D3 [1,25-(OH)2D3], by initiating a cascade of molecular events leading to bone mineralization and remodelling and by modulating cell growth and differentiation [37]. RARs mediate the biological effects of retinoids, derivatives of vitamin A, which are required for cellular growth and development [38]. In particular, RAR-γ is known to be involved in correct bone development during the early developmental stages [39]. So in this instance, we used VDR-α and RAR-γ gene expression as biomarker for backbone calcification.

Concerning sexual maturation, GnRH mainly orchestrates vertebrate puberty and gametogenesis. In the two GnRH-form zebrafish species, GnRH3 has been shown to elicit gonadotropin-releasing activity and is thought to assimilate non-redundant functions of GnRH1 [40]–[42]. To understand whether the presence of L. rhamnosus could influence gonad development and sex differentiation through the GnRH pathway, we used a GnRH3-GFP transgenic line [43]; in parallel, the ongoing backbone calcification was determined in larvae. Zebrafish is a juvenile protogynous hermaphrodite species [44] that, irrespective of their genetic gender, first develop ovary-like gonads. Bisexual differentiation takes place when protogynous ovaries of some specimens in a population undergo differentiation, turning first into an intermediate phase termed ‘altered ovary’ and then into testes [45]. To determine whether L. rhamnosus could act on the onset of gonad differentiation, we timed the appearance of the first testis. The progress of gonad maturation by histological analysis and the sex ratio were registered at the end of the experiment.

Upon the presence of L. rhamnosus, expression of genes related to growth and development increased and enhanced GnRH3 expression was observed; moreover, an earlier onset of backbone calcification and gonadal differentiation were found, together with significant changes in the sex ratio of the final populations.

## Results

### Morphometric Results

**Figure 1:**
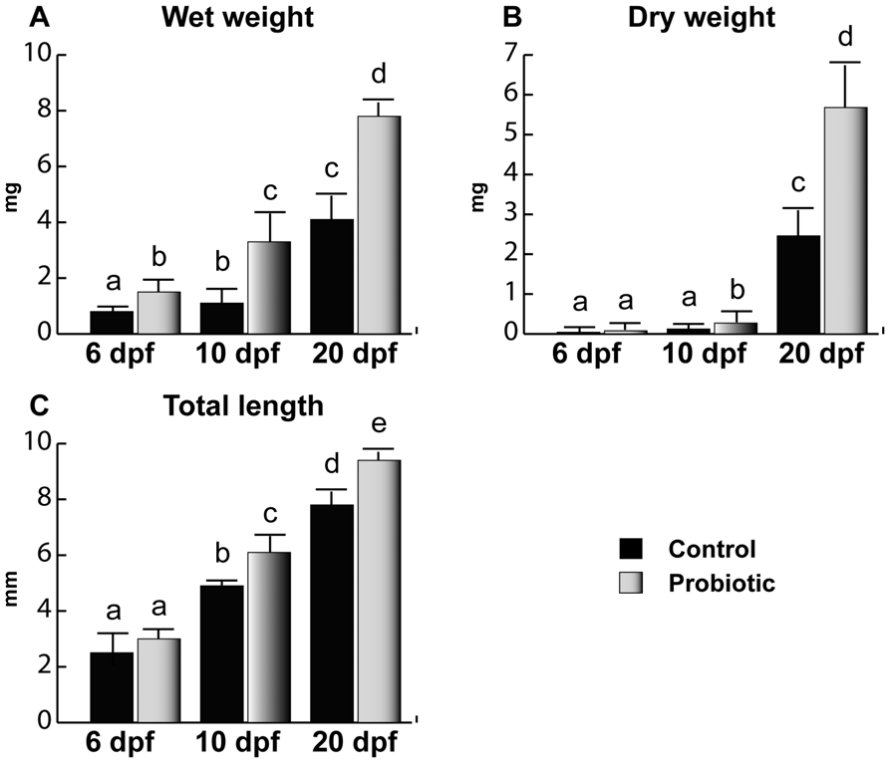
Body weight and total length. Wet weight (A), dry weight (B) and total length (C) of zebrafish larvae from control (n = 10) and probiotic (n = 10) groups and collected at days 6, 10 and 20 post fertilization (3 replicates). Values with different superscript letters are significantly different (P<0.05).

L. rhamnosus administration increased both weight (Figure 1A and B) and total length (Figure 1C). No significant difference was observed at 6 dpf for dry weight and total length, while a higher wet weight was found in probiotic fed larvae body weight and total length. At 10 dpf, a significant increase in length and body weight was observed in the probiotic fed larvae compared to control. A further increase in size was observed at 20 dpf in fingerlings treated with L. rhamnosus compared to the control.

### Molecular Results

#### Retention of L. rhamnosus in zebrafish larvae and juveniles

Zebrafish gastrointestinal tracts retained L. rhamnosus during the entire chronic administration. In the control group, no L. rhamnosus was detected, whereas in the probiotic group, presence of L. rhamnosus was detected in both larval and juvenile stages (Table 1).

**Table 1:** Lactobacillus rhamnosus retention.

Sampling point	Control		Probiotic	
	Min copy number	Max copy number	Min copy number	Max copy number
6 dpf	Undetected	<0.1	3.87	80.72
10 dpf	Undetected	<0.1	0.84	9.62
20 dpf	Undetected	<0.1	100.46	701.37
3 wpf	Undetected	<0.1	140.99	6493.59
6 wpf	Undetected	<0.1	128.20	44834.94
9 wpf	Undetected	<0.1	22.67	2712.39

### Stress Biomarkers

**Figure 2:**
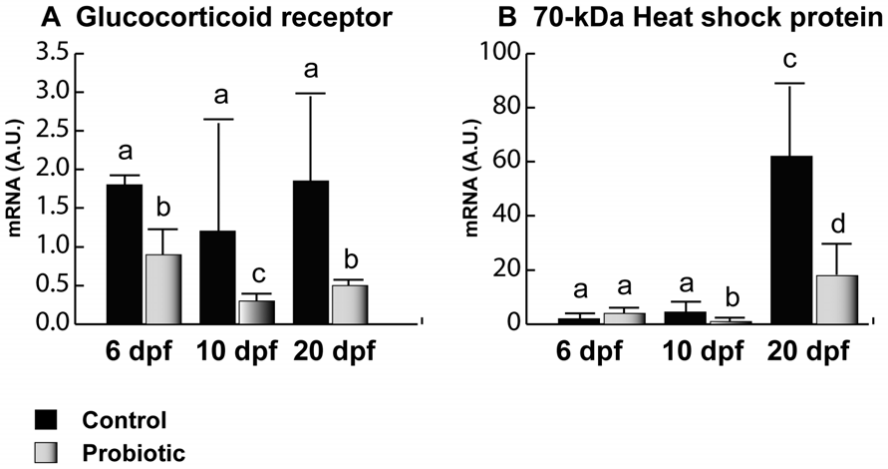
Stress biomarkers. Relative GR (A) and HSP70 (B) gene expression in zebrafish larvae from control (n = 10) and probiotic (n = 10) groups and collected at days 6, 10 and 20 post fertilization (3 replicates). Values with different superscript letters are significantly different (P<0.05).

Concerning GR gene expression (Figure 2A), no significant differences were evaluated among the different developmental stages in the control; however, in the probiotic group, significantly lower gene expression levels were observed at 6, 10 and 20 dpf. Concerning HSP70 (Figure 2B), an increased gene expression trend was found in the control group from day 6 to 20 pf. In particular at 6 dpf, no significant differences were found between control and probiotic groups. At 10 and 20 dpf, significantly lower gene expression levels were registered in the probiotic treated group compared to the control.

### Muscle Growth Biomarkers

**Figure 3:**
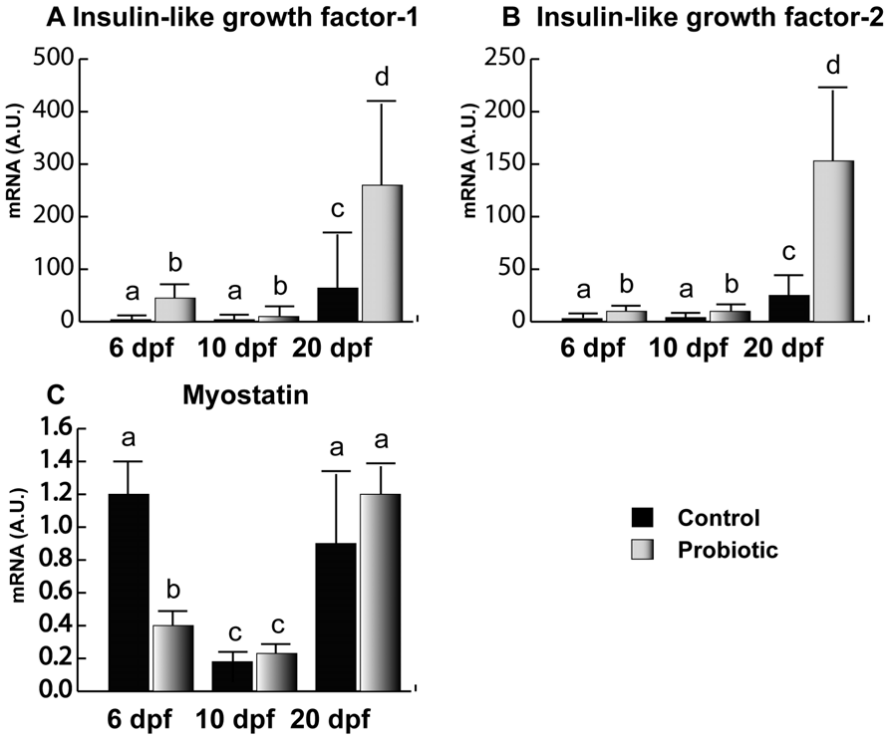
Muscle growth biomarkers. Relative IGF-1 (A), IGF-2 (B) and MSTN (C) gene expression in zebrafish larvae from control (n = 10) and probiotic (n = 10) groups collected at days 6, 10 and 20 post fertilization (3 replicates). Values with different superscript letters are significantly different (P<0.05).

IGF-1 (Figure 3A) and IGF-2 (Figure 3B) showed very similar gene expression trends. In the control, IGFs gene expression was almost undetectable until day 20 pf. On the contrary, in probiotic group, IGF-1 and -2 gene expressions were clearly detectable as early as 6 dpf. In addition, IGFs gene expression levels were significantly higher in the probiotic group in all the developmental stages analyzed (6, 10 and 20 dpf) Concerning MSTN (Figure 3C), at 6 dpf, the probiotic group showed lower levels of gene expression with respect to the control. The lowest levels of MSTN were found at 10 dpf in both control and probiotic groups without any significant difference between the two experimental groups. No significant differences between the two experimental groups were found at 20 dpf either.

### General Development Biomarkers

**Figure 4:**
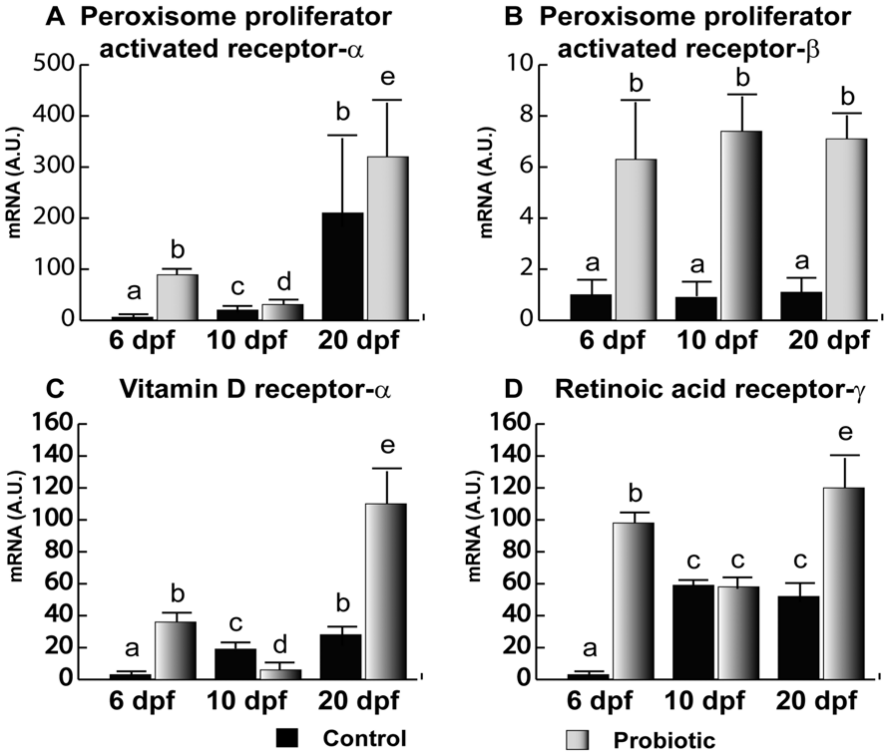
General development biomarkers. Relative PPAR-α(A), PPAR-β (B), VDR-α (C) and RAR-γ (D) gene expression in zebrafish larvae from control (n = 10) and probiotic (n = 10) groups collected at days 6, 10 and 20 post fertilization (3 replicates). Values with different superscript letters are significantly different (P<0.05).

Analogously to what was observed for IGFs, PPAR-α gene expression (Figure 4A) in the control group was almost undetectable until 10 dpf. In contrast, at 6 and 10 dpf, the probiotic group showed higher gene expression levels. At 20 dpf, no significant differences were evaluated. Of note, gene expression levels observed in the probiotic group at 6 dpf were even significantly higher than the levels observed in the control group at day 10 pf. Also, PPAR-β (Figure 4B) was modulated by probiotic treatment; in fact, at 6, 10 and 20 dpf, higher gene expression levels were found in the probiotic group. However, PPAR-β gene expression did not seem to vary during development. Concerning VDR-α (Figure 4C), while at 6 dpf VDR-α gene expression in the control group was almost undetectable, a significantly higher level was found in the probiotic group. This level was even significantly higher than the VDR-α gene expression levels registered at 10 dpf in both control and probiotic groups. Moreover, it is noteworthy that VDR-α gene expression level in the probiotic group at 6 dpf was similar to the value registered at 20 dpf in the control group. At 20 dpf, VDR-α gene expression was significantly higher in the probiotic group with respect to control group.

At 6 dpf, RAR-γ (Figure 4D) gene expression was almost undetectable in the control group. On the contrary, at 6 dpf in the probiotic group, a significantly higher gene expression was detected; the aforementioned level was even significantly higher than the levels at 10 dpf (in both control and probiotic groups) and 20 dpf in the control group. At 20 dpf, gene expression was significantly higher in the probiotic group with respect to control group (Figure 4D).

### GnRH3 Neuronal Development

**Figure 5:**
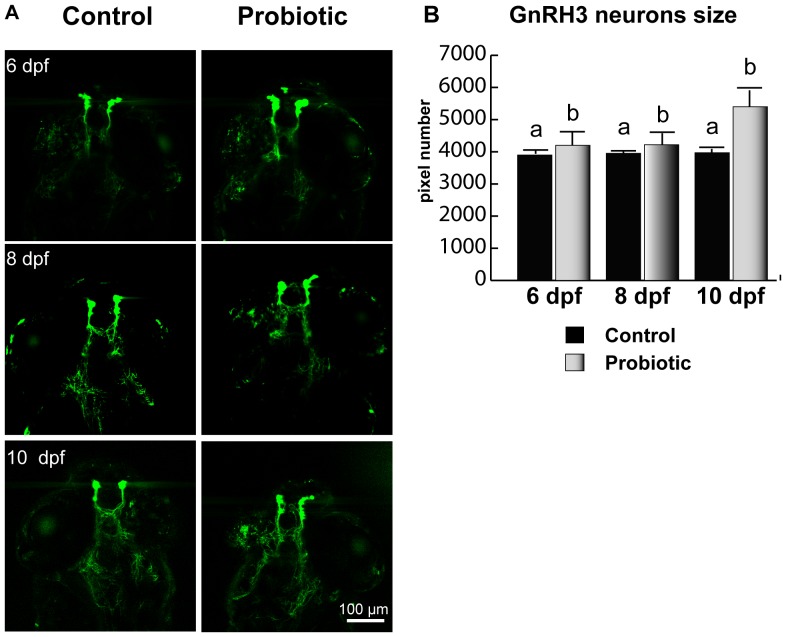
GnRH3 neurons soma size. GnRH3 neurons in transgenic zebrafish larvae (n = 10) sampled at 6, 8 and 10 dpf (A). Assay was conducted in triplicate. On the right, GnRH3 neurons size (pixels) (B). The green channel of RGB images collected at magnification of 20X (whose some representative are shown here) was extracted, converted to grayscale and GnRH3:EGFP neurons were analyzed for size using the “Analyze > Analyze particles…” macro. We set up the macro-assisted algorithm to exclude the background pixelation. At 6, 8 and 10 dpf, 10 fish were analyzed for each experimental condition. Values with different superscript letters are significantly different (P<0.05).

Neuronal development was monitored using a GnRH3 transgenic zebrafish [43]. Briefly, a 2.4 kb sequence upstream of the gonadotrophin-releasing hormone (GnRH3) decapeptide coding region including 1.3 kb of 5′ flanking sequence, exon I, intron I and part of exon II was cloned and inserted into an expression vector p-EGFP1 that includes an EGFP coding sequence. Linearized construct was microinjected into the cytoplasm of zebrafish embryos at the one-cell stage. The number of pixels inside GnRH3 neurons increased in larvae fed on probiotic. In all the stages analyzed (6, 8 and 10 dpf), higher pixel numbers in GnRH3 neuron were registered in the probiotic group. We relate this finding to the elevated GnRH3 expression, which increased upon L. rhamnosus administration. From 8 to 10 dpf, larvae from control group did not show any significant change; on the contrary, at all time points analyzed, fish treated with probiotic revealed significantly higher pixel number with respect to control (Figure 5A).

### Backbone Calcification

**Figure 6:**
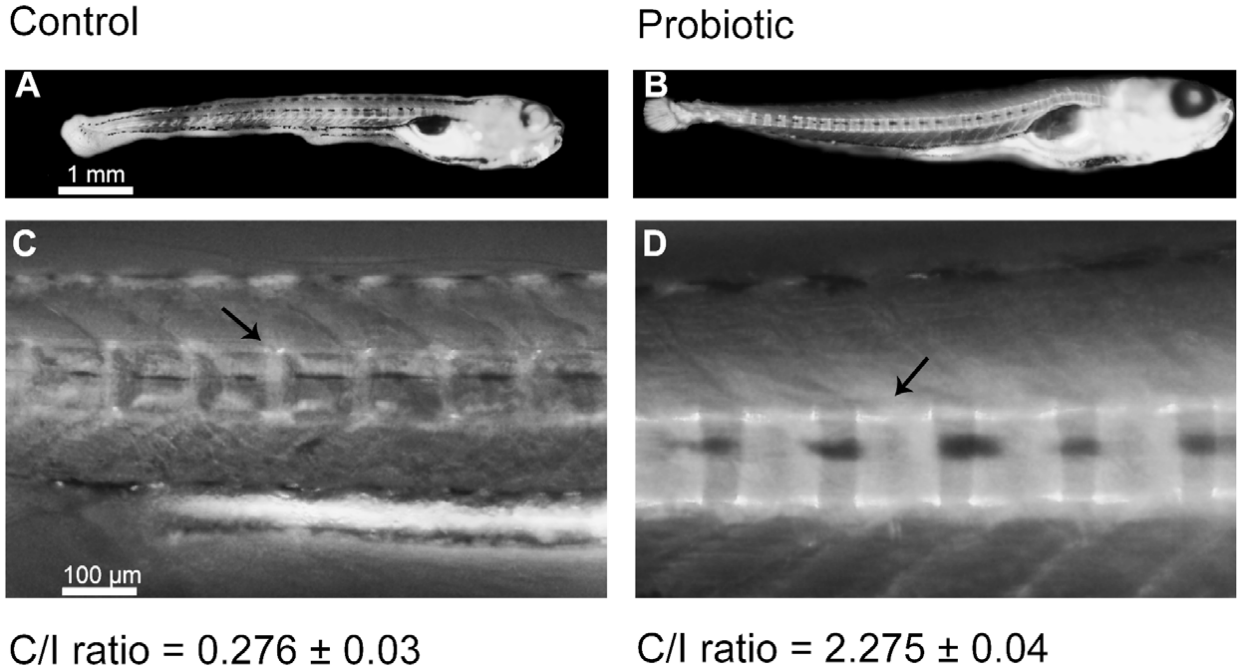
Backbone calcification. Two representative pictures showing backbone calcification of zebrafish larvae at 12 days post fertilization (dpf) from control (A, n = 10) and probiotic group (B, n = 10) respectively (3 replicates). 10× magnification of the centra (black arrows) and the Centra/Intracentra ratio with average values are reported (C and D).

Backbone centra of the probiotic-treated larvae exhibited more extensive calcification (Figure 6B) than the centra from control group’s larvae (Figure 6A). In fact, the Centra/Intracentra ratio (C/I –see materials and methods section for further description) from probiotic group was significantly higher with respect to control (2.275±0.04– Figure 6D vs. 0.276±0.03– Figure 6C respectively), which indicated faster backbone calcification in the probiotic treated zebrafish larvae.

### Gonad Differentiation and Sex Ratio

**Figure 7:**
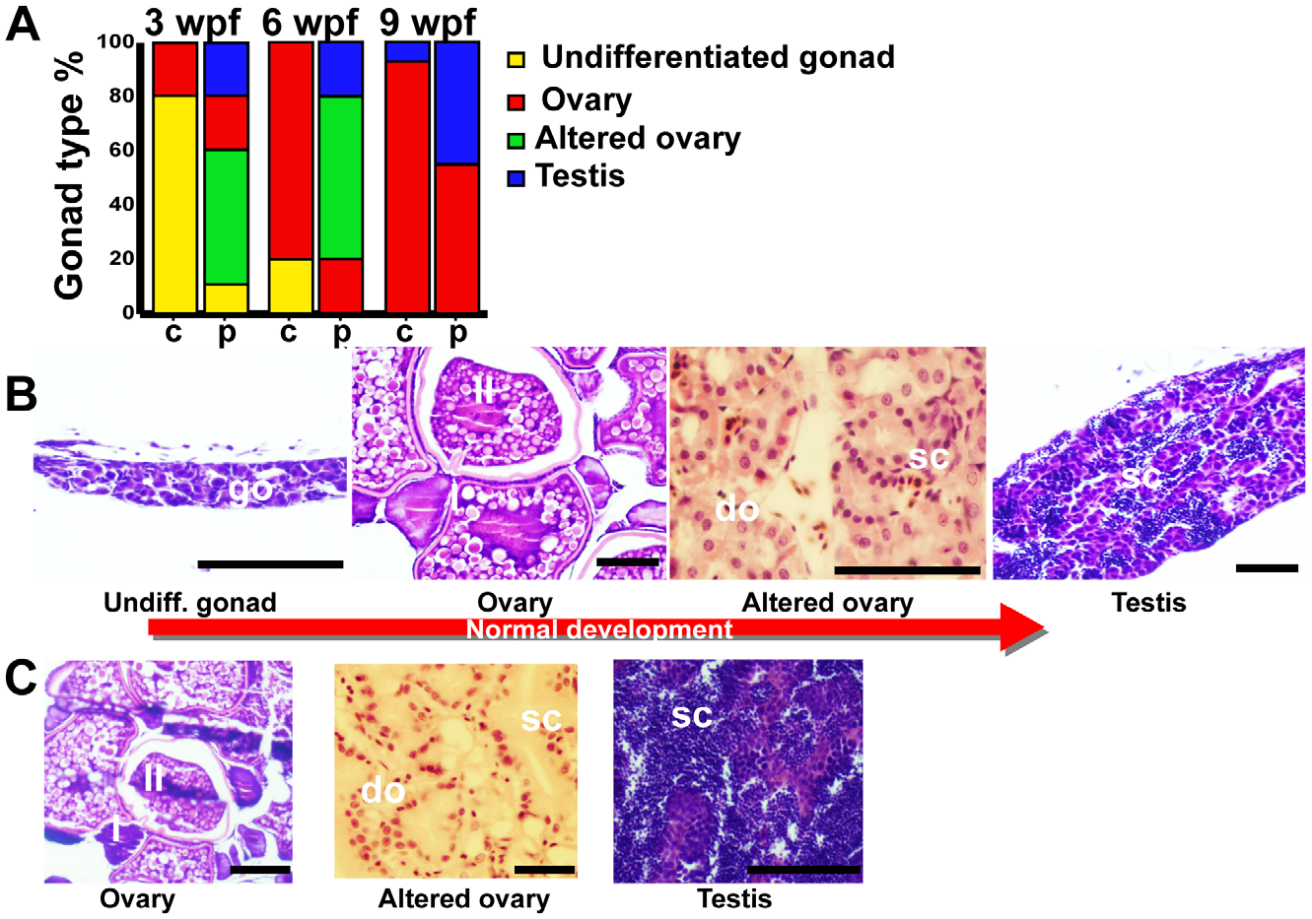
Gonad development. A: Gonad type percentage of 10 zebrafish from control (c) and probiotic (p) groups at 3, 6 and 9 weeks post fertilization – wpf. Assay was run in triplicate. B: Normal gonad development; progression from undifferentiated gonad, to ovary, altered ovary and testis. C: representative pictures of ovary, altered ovary and testis prematurely observed in probiotic treated fish at 3 weeks post fertilization. Legend: go, gonocyte; do, degenerating oocytes; sc, spermatocyst; I, stage I oocyte; II, stage II oocyte; scale bars 50 µm.

L. rhamnosus administration significantly affected both the timing of gonad differentiation and the population sex ratio. At 3 weeks pf, in the control group, only 20% of specimens showed ovary-like gonads, while the majority of fish still showed undifferentiated gonad (Figure 7). In the probiotic group, only 10% showed undifferentiated gonads, whereas 20% of specimens had ovary-like gonads, 50% showed altered ovaries and 20% already presented testis. At 6 weeks pf (Figure 7), 80% of specimens from the control group showed ovary-like gonads and the remaining 20% of fish showed still undifferentiated gonad. In the probiotic group the 20% showed ovary-like gonads, 60% presented altered ovaries and 20% presented testis. At 9 weeks pf (Figure 7), 93% of fish from the control group were females and only 7% males. In the probiotic group, 55% were females and 45% males. No sexual behavior was observed until 10 weeks pf in both control and probiotic groups.

## Discussion

Modulation of host microbial community is usually associated with host physiological changes [46]. In our study, zebrafish raised on a L. rhamnosus-supplemented diet showed faster development, with earlier onset of backbone calcification and gonad differentiation and a drastic change in the sex ratio in the final population.

Previous studies [46]–[48] showed that beneficial microbes led an organism to more efficiently use diet-derived energy sources, leading to growth improvement [49]. This is a likely explanation for the increase of larval size we observed in our study. Rawls and co-workers [9] demonstrated that microbiota could modulate the expression of 212 genes, 8 of which were involved in promotion of nutrient metabolism. Analogously to what we observed in our previous work [50], in the present study, L. rhamnosus treatment influenced the gene expression level of the entire set of genes analyzed in a very similar fashion to what observes in the false percula clownfish, which make us infer a conserved physiological response to L. rhamnosus treatment. However here we find that L. rhamnosus can stimulate an organism developmental profile. It was not surprising that the lower physiological and cellular stress levels registered (GR and HSP70) coincided with an increase in growth. At the same time, growth-related factors (IGF-1 and IGF-1I) showed higher levels in treated fish, while MSTN levels were significantly reduced. We hypothesized that because glucocorticoids are able to inhibit IGF-1 [51] and to increase MSTN gene expression in muscle cells [20], lower levels of GR gene expression yielded higher growth through IGF-signaling pathways.

Vitamin D and retinoic acid are essential molecules for morphogenesis and chondrogenesis [32], [52], [53]. IGFs and vitamin D play the role as main determinants of backbone calcification and bone mass accretion, the former controlling muscle and skeletal cell growth and division, the latter stimulating calcium absorption and retention [54]. We relate the faster calcification observed in the treated larvae with the upregulation of IGF-I and –II levels and with a plausible increased availability of LAB metabolites, as the gene expression levels of these markers revealed.

Several nuclear receptors mediate the positive action of free fatty acids and vitamins, which are compounds that are naturally delivered by LAB [55]–[57]. PPAR-α and β, RAR-γ and VDR-α were all increased by L. rhamnosus. Lactobacillus fermentation produces short chain fatty acids, which are known to activate PPARs [29]; in agreement with previous data on mice [48] the higher PPARs gene expression recorded could be attributed to the supplied probiotic activity in vivo. Furthermore, it is known that PPAR-α and β are involved in skeletal development, lipid metabolism and cell proliferation [30]. Here we speculate a positive correlation between these systems and the accelerated development registered. Our observations agree with previous studies that report probiotics affecting development, specifically acting on bone accretion [58], [59]. We hypothesize that L. rhamnosus supplementation permits the recipient to develop faster in the following manner : 1) through the delivery of molecules such as fatty acids and vitamins as supported by the PPARs, VDR-α and RAR-γ gene expression increases and the increased ability to metabolize nutrients ensues; 2), through stimulation of the IGF system. It is reported that GnRH pulses amplitude increase together with an increase of gonadal sex steroid output, which in turn lead to higher levels of IGF-1 and vitamin D production [54]. We hypothesize that, by positive feedback, IGF-1 further stimulates the secretion of sex steroids, which in turn potentiated backbone calcification. This could also have influenced the timing of sex differentiation. We assume that the larger GnRH3 neurons size corresponded to a higher GnRH3 expression; this could be related to the higher gene expression levels observed for VDR and IGFs, which in turn could accelerate larval development in terms of growth, backbone calcification and sex differentiation. Zebrafish have two GnRH forms, where GnRH3 has been suggested to elicit gonadotropin-releasing activity [60] and is thought to play a role in sex differentiation [61]. In fact, it was reported that partial ablation of GnRH3 neurons leads to an increased percentage of female fish, while the total ablation leads to an all female population [62]. Sex determination occurs in zebrafish between 21 and 23 dpf [63]. The modulation of GnRH3 observed in probiotic-treated zebrafish could be related to the noteworthy earlier gonad development observed in probiotic-treated fish and also to the sex ratio registered at 9 weeks.

In conclusion, we report that adding L. rhamnosus to the natural zebrafish microflora -from birth to sexual maturity- results into a faster backbone calcification and gonadal differentiation, with altered sex ratios. In the current study we further report that L rhamnosus can also affect the timing of sex differentiation and lead to significantly different sex ratios. Some literature reports highly variable sex ratios in zebrafish populations [64], in fact the unexpected sex ratio found in the control group (over 90% were females) was previously described but we consider noteworthy that the siblings’ population treated with L. rhamnosus developed such a different sex ratio (55% females).

Additional in vitro and in vivo studies should focus on deciphering the signals delivered by L. rhamnosus that have a direct effect on backbone calcification and gonadal development. Further studies are also needed to identify how probiotic administration is involved in sex ratio modulation. These analyses might provide a list of candidate pathways through which the microbiota could affect the physiology of development.

## Materials and Methods

### Ethics Statement

Wild type and GnRH3-GFP transgenic zebrafish larvae (n = 110, 3 replicates) between the ages of 0 and 20 dpf and juveniles (n = 35, 3 replicates) between the ages of 3 weeks pf and 9 weeks pf were euthanized with an overdose of MS-222 (2 g/l). All procedures were approved by the Institutional Animal Care and Use Committee at the University of Maryland (IACUC Protocol # AP090209-01).

**Table 2:** Primers sequences.

Gene	Sequence
GR	Fwd: 5′–GGCCAGTTTATGCTTTTCCA–3′
	Rev: 5′–CTTCCGCAAGTGAGAACTCC–3′
HSP70	Fwd: 5′–AAAGCACTGAGGGACGCTAA–3′
	Rev: 5′–TGTTCAGTTCTCTGCCGTTG–3′
IGF-1	Fwd: 5′–GGCAAATCTCCACGATCTCTAC–3′
	Rev: 5′–GACTCTCTCTGTTCTCTTTGGC–3′
IGF-2	Fwd: 5′–GAGTCCCATCCATTCTGTTG–3′
	Rev: 5′–GTTTACCGTTGTTTGTTTCCT–3′
MSTN	Fwd: 5′–GGACTGGACTGCGATGAG–3′
	Rev: 5′–GATGGGTGTGGGGATACTTC–3′
PPAR-α	Fwd: 5′–TCCACATGAACAAAGCCAAA–3′
	Rev: 5′–AGCGTACTGGCAGAAAAGGA–3′
PPAR-β	Fwd: 5′- CAGGTGACGCTGCTGAAATA-3′
	Rev: 5′- CGGAGGAACTCTCTCGTCAC-3′
VDR-α	Fwd: 5′- CTCCAGTGAGGAGGATCAGC-3′
	Rev: 5′- TCTTCAGCCGTCAGGTCTCT-3′
RAR-γ	Fwd: 5′- ATTCCGCCAGAGAGCTATGA-3′
	Rev: 5′- TAGGCCCAGGTCTAGCTGAA-3′
L. rhamnosus	Fwd: 5′- CCCACTGCTGCCTCCCGTAGGAGT-3′
	Rev: 5′- TGCATCTTGATTTAATTTTG-3′

### Animals and Experimental Design

Adult GnRH3-GFP transgenic zebrafish [43] pairs were spawned individually and larvae were raised under a 12∶12 h light/dark cycle at 28°C. Starting from 3 days post-fertilization (dpf), larvae were given Paramecia twice daily. In the control group, zebrafish larvae were fed a standard diet (which consisted of Paramaecia from 3 to 12 dpf, Artemia nauplii from 13 to 21 dpf and dry food from 22 dpf), while in the probiotic group, L. rhamnosus IMC 501®, generously provided by Synbiotec Srl, (Camerino, Italy) was delivered via live food as previously described [65] and added to the water at a final concentration of 10^6^ CFU bacteria ml^−1^. Treatments were carried out for 10 weeks, until puberty. The control and treated groups (200 larvae each) were tested in triplicate. Each 200-embryo cluster was collected from a different zebrafish pair. From each experimental group:

- Fish were sampled and stored at −80°C at 6, 10 and 20 dpf [according to 9] for Real time PCR analyses and 10 fish were euthanized and collected for morphometric analyses (total length, wet and dry weight).
- Fish were sampled at 6, 8 and 10 dpf for the GnRH3 neurons development study under confocal microscope.
- Fish larvae were sampled for the valuation of backbone calcification at 12 dpf -when the onset of vertebral calcification is expected [12]- under fluorescent microscope.
- Fish were sampled at 3, 6 and 9 weeks post fertilization (pf) and fixed in paraformaldehyde (PFA) 4% for the histological sections and evaluation of gonadal development.
- Fish were collected for L. rhamnosus intestinal retention.

### Morphological Studies

A pool of 10 larvae was weighted immediatly upon sampling to register wet weight (WW) and the same pool were subsequently lyophilized using a Labconco Freeze dry system to obtain dry weight (DW). WW and DW were recorded using the balance Mettler UMT2 microbalance accurate to 0.1 µg. Total length (TL), backbone calcification and GnRH3 neuron size- were performed with the use of bright field fluorescence microscopy, using a Zeiss Axioplan 2 microscope (Carl Zeiss MicroImaging, Inc., Thornwood, NY, USA) with appropriate filters, an Attoarc HBO100 W power source and an Olympus DP70 digital camera (Olympus, Tokyo, Japan). GnRH3 neuron development was observed under confocal microscopy, using a Zeiss Radiance 2100 laser scanning system together with Laser-Sharp and LSM imaging software (Carl Zeiss MicroImaging, Inc.). All confocal pictures are of extended focus views. Determining the localization of GnRH3 perikarya and fibres to specific regions was performed in accordance with a previous study [43]. The average size of GnRH3 neurons was determined with an unbiased method, by measuring pixels inside the neuron with Image J software as follows. After collecting RGB images at magnification of 20X and delimiting the area which included the GnRH3 neuron, the green channel was extracted and converted to grayscale (“Process > Binary > make binary”) and the selected GnRH3: EGFP neuron was analyzed for size using the “Analyze > Analyze particles…” macro. We set up the macro-assisted algorithm to exclude the background pixilation as follows: Size (Pixel) 200-Infinity, Circularity 0.00–1.00. At 6, 8 and 10 dpf, 10 fish were analyzed for each experimental condition.

### L. rhamnosus Intestinal Retention

Zebrafish individuals were collected at 6, 10, 20 dpf and at 3, 6 and 9 weeks pf and washed twice for 5 min each using water filtered through a MilliQ UV Plus water purification system (Millipore, Bellerica, MA) Samples were then placed into 95% ethanol and stored until the end of the timecourse. Gut were sampled for DNA extraction, the samples were homogenized using an Ultra Turrax T8 (IKA, Wilmington, NC) with a 5 mm generator in 500 µl of 0.1 M ethylenediamine tetraacetic acid, 0.5% sodium dodecyl sulfate for 30 ss at a power of 4. 50 µl of 20 mg/ml proteinase K (Sigma, St. Louis, MO) was added and mixed by inversion. Samples were incubated for 18 h at 55°C. 82.5 µl of 5 M NaCl and 82.5 µl of 10% acetyltrimethyl ammonium bromide, 0.7 M NaCl was added and mixed by inversion. The samples were incubated at 55°C for 10 min. 500 µl of chlorophorm was added to each sample and mixed. The samples were incubated at room temperature for 10 min and then spun at 3000×g for 15 min to separate the phases. The aqueous phase was transferred to a new plate. The DNA was then column purified using a Zymo Research ZR-96 clean and concentrate -5 kit according to the manufacturer's directions (D4024, Irvine, CA) The resultant nucleic acids were checked for purity and quantified using a Nanodrop ND-1000 spectrophotometer (Wilmington, DE). 10 ng of DNA from each sample was amplified with primers in Table 2 at 500 nM each using syber green containing iTaq 2X Supermix from Bio-Rad with ROX standard (172-5850, Hercules, CA). Cycling conditions consisted of an initial denaturation of 95°C for 2 min followed by 40 cycles of 95°C for 15 s, 60°C for 30 s and 72°C for 1 min with data collection occurring at the 72°C elongation. Data collection and analysis was performed using 7500 Fast Real Time PCR system and its associated software from Applied Biosystems (Carlsbad, CA). The reaction was terminated with a melting curve analysis to determine product purity. A standard curve was included as a positive control and to determine reaction efficiency. No template containing reactions were included as negative controls. Baseline and threshold values were determined automatically using the software and confirmed manually. Resultant cycle threshold (CT) values were compared to the controls to determine the presence of L. rhamnosus.

### Gene Expression Analysis

Minikit RNAeasy® (Qiagen) extraction kit was used for total RNA extraction from whole larval body following the manufacturer’s protocol. Total RNA extracted was eluted in 15 µL of RNAse-free water. Final RNA concentrations were determined by nanodrop and the RNA integrity was verified by ethidium bromide staining of 28S and 18S ribosomal RNA bands on a 1% agarose gel. RNA was stored at −80°C until use. Total RNA was treated with DNAse (10 UI at 37°C for 10 min, MBI Fermentas). A total amount of 5 µg of RNA was used for cDNA synthesis employing 0.5 µg oligod(T)+adapter primer, 5′-GACTGCAGTCGACATCGATTTTTTTTTTTTTTTTTT-3′, in a buffer containing 50 mM Tris-HCl (pH 8.3), 75 mM KCl, 3 mM MgCl2, 10 mM DTT, 0.5 mM of each dNTP, 40 units RNAse OUT (Invitrogen) and 200 units of Superscript II RT (Invitrogen, Life Technologies, Milan, Italy). Cycling conditions were 70°C for 5 min, 42°C for 52 min and 72°C for 15 min.

Triplicate PCRs were carried out for each sample analyzed. After optimization (primer annealing temperature and cDNA dilutions) the PCRs were performed with the SYBR green method in 7500 Fast Real time PCR system from Applied Biosystems (Carlsbad, CA). The reactions were set up in a 96-well plate by mixing, for each sample, 1 µL of diluted (1/20) cDNA, 5 µL of 2X concentrated SYBR Green PCR Master Mix (BioRad) containing SYBR Green as fluorescent intercalating agent, 0.3 µM forward primer and 0.3 µM of reverse primer. The thermal profile for all reactions was 15 min at 95°C, 45 cycles of 20 s at 95°C, 20 s at 60°C, 20 s at 72°C. The fluorescence monitoring occurred at the end of each cycle. Additional dissociation curve analysis was performed and showed in all cases a single melting curve. A relative quantification of cDNA was made using β-actin (β-Act) as housekeeping gene. Pfaffl’s mathematical model [66] was applied to determine the ratio between the different expression of the target gene (GR, HSP70, IGF-1, IGF-2, MSTN, PPAR-α and PPAR-β, VDR-α, RAR-γ) in the treated and control group and the different expression of the standard gene (β-Act) in the treated and control group.

Modification of gene expression is represented with respect to the control, which is assumed to have the value of 1 A.U. (arbitrary unit). Using the Pfaffl’s mathematical model, the cDNA levels of three genes were quantified in each group and at each time of sampling. To test Real time PCR efficiency, serial dilutions of cDNA, each in triplicate, were amplified by Real time PCR using the specific primers for target genes and the standard gene. Table 2 shows primers sequences used in the present study (at a final concentration of 10 pmol/µl).

### Backbone Calcification - Calcein Immersion

Backbone development and calcification were visualized as previously described [67]. Immersion solutions (0.2%) were prepared by dissolving 2 g of calcein powder (Sigma Chemical, St. Louis, MO) in 1 liter of deionized water. Due to calcein’s strong acidifying affects, an appropriate amount of NaOH (0.5 N) was added to the solution to restore the pH to its original value. Zebrafish larvae to be treated were netted and immersed in the solutions in petri dishes for 10 min. After the immersions, the larvae were rinsed 3 times in fresh water and then allowed to stand for 10 min to allow the excess, unbound calcein to diffuse out of the tissues. The larvae were euthanized in tricaine–methanesulfonate (MS-222, Sigma-Aldrich) and mounted on glass slides with methyl-cellulose (3%). Observations were carried out by using an Olympus BX60 microscope with a green fluorescence filter set. Images (84,882 pixels) were captured with a Quatrix (Photometrics, Inc.) CCD camera and compatible IPLab 3.0 (Scanalysis, Inc.) software and saved in TIFF format. Composite images of the larvae were produced with Adobe PhotoShop 5.0 and saved in MS-ppt format. The ratio between the length of the vertebral bodies (centra) and the space between each centra were named Centra/Intracentra ratio (C/I). C/Is were registered in every single specimen and the C/I averages for each larva were determined. Successively, C/I averages from 10 zebrafish larvae, respectively, from control and probiotic groups were evaluated and compared.

### Histology Study

Larvae at 3, 6 and 9 weeks post-fertilization were fixed by immersion in PFA 4% solution for 24 h, dehydrated in increasing concentrations of ethanol and embedded in paraffin wax. With use of standard histochemical techniques, sagittal sections of 5-µm thickness were stained with hematoxylin/eosin. Sections were examined under light microscope to detect gonad maturation and differentiation.

### Statistical Analysis

Results were expressed as the mean ± s.d. The significance of differences was determined using a one-way ANOVA, followed by Tukey’s test for multigroup comparisons, using statistical software package, SigmaStat 3.1 (Systat software Inc.). A P value <0.05 was regarded as statistically significant.
